# Bi-objective integer programming for RNA secondary structure prediction with pseudoknots

**DOI:** 10.1186/s12859-018-2007-7

**Published:** 2018-01-15

**Authors:** Audrey Legendre, Eric Angel, Fariza Tahi

**Affiliations:** IBISC, Univ Evry, Université Paris-Saclay, Evry, 91025 France

**Keywords:** RNA, Secondary structure, Pseudoknot, Integer programming, Bi-objective, Optimal solutions, Sub-optimal solutions

## Abstract

**Background:**

RNA structure prediction is an important field in bioinformatics, and numerous methods and tools have been proposed. Pseudoknots are specific motifs of RNA secondary structures that are difficult to predict. Almost all existing methods are based on a single model and return one solution, often missing the real structure. An alternative approach would be to combine different models and return a (small) set of solutions, maximizing its quality and diversity in order to increase the probability that it contains the real structure.

**Results:**

We propose here an original method for predicting RNA secondary structures with pseudoknots, based on integer programming. We developed a generic bi-objective integer programming algorithm allowing to return optimal and sub-optimal solutions optimizing simultaneously two models. This algorithm was then applied to the combination of two known models of RNA secondary structure prediction, namely MEA and MFE. The resulting tool, called BiokoP, is compared with the other methods in the literature. The results show that the best solution (structure with the highest F_1_-score) is, in most cases, given by BiokoP. Moreover, the results of BiokoP are homogeneous, regardless of the pseudoknot type or the presence or not of pseudoknots. Indeed, the F_1_-scores are always higher than 70% for any number of solutions returned.

**Conclusion:**

The results obtained by BiokoP show that combining the MEA and the MFE models, as well as returning several optimal and several sub-optimal solutions, allow to improve the prediction of secondary structures. One perspective of our work is to combine better mono-criterion models, in particular to combine a model based on the comparative approach with the MEA and the MFE models. This leads to develop in the future a new multi-objective algorithm to combine more than two models. BiokoP is available on the EvryRNA platform: https://EvryRNA.ibisc.univ-evry.fr.

## Background

RNAs are involved in numerous pathologies such as cancer and neurodegenerative diseases. Determining the structure of an RNA is an important step in the understanding of its biological and biochemical function, its classification and its interaction with other molecules. In this paper, we are interested in the prediction of the secondary structure of RNAs with pseudoknots. Pseudoknots can have important roles in the translation process. For example, some studies have shown that the interaction of a pseudoknot with the ribosome induces a break of the ribosome during the translation, by causing a deformation of the tRNA in the P site [1].

Predicting the secondary structure with pseudoknots of an RNA sequence is a subject which is heavily studied in the literature. In fact, this problem was proved to be NP-hard for various energy models [2, 3] and, as the current provided tools are not satisfactory, it is still an open subject. Two main approaches exist for predicting RNA structures (with or without pseudoknots): the thermodynamic approach and the comparative approach. The thermodynamic approach consists in, either computing the structure of minimum free energy (MFE) according to a set of thermodynamic parameters, or computing the structure of maximum expected accuracy (MEA) with a partition function. The comparative approach consists in finding a conserved RNA structure between several species. This approach needs therefore several (homologous) sequences as inputs, unlike the first approach where only one sequence is needed.

Many tools have been proposed in the literature for predicting RNA pseudoknots. We can cite for instance tools based on MFE models [4–9], tools based on MEA models [10, 11] and tools based on the comparative approach [12, 13]. However, the results of a single given model can only approach the real structure. For example, it is now established that the real structure has a very low energy, but not necessarily the minimum one (indeed, many factors are involved, such as the environment). Approaches able to combine different models are therefore interesting. To our knowledge, very few tools have been proposed to combine different models for the prediction of secondary structures of RNAs with pseudoknots. Combination has been used for the prediction of a consensus structure of several homologous sequences, as performed in ILM [13] which combines the comparative approach with an MFE model, and in IPknot [10] which combines the comparative approach with an MEA model. An algebraic dynamic programming method [14] has also been proposed to combine the MEA and the MFE models. However, no dedicated tool is available. Moreover, very few tools, namely pKiss [4], McGenus [5] and Tfold [12], have been proposed to return several solutions of secondary structures with pseudoknots. Proposing a unique solution, the optimal one according to a given model, is restrictive, for the reasons given above. It is important to consider also sub-optimal solutions. Our goal is to develop a method combining different models and returning both several optimal and several sub-optimal solutions. In this paper, we are interested in the thermodynamic approach, as we consider a single RNA sequence of interest as input.

The majority of RNA secondary structure prediction tools were developed using the dynamic programming methodology [4, 5, 7, 11]. In [6] and [10], another approach was proposed: integer programming. An integer program is a mathematical formalization of a problem. It consists in an objective function to optimize on a set of integer variables, subject to a set of linear constraints. This approach is very flexible, allowing to model mathematically a large range of problems. It has been applied to various domains, from economy to industry. To our knowledge, only one team has used integer programming for RNA secondary structure prediction with pseudoknots. First they developed an integer program [6] to find the structure of MFE using the stacking energy parameters of Mfold 3.0 [15]. Then they provided the IPknot software [10] based on an MEA model using base pair probabilities computed with different models like the McCaskill [16] or the Dirks and Pierce [8] models. This team also used integer programming to predict RNA-RNA interactions [17]. Note that integer programming has also been employed in related domains such as multiple RNA sequence-structure alignment [18] or 3D RNA structure by inserting local 3D motifs in RNA secondary structure [19].

In this paper, we propose an original method based on bi-objective integer programming minimizing two criteria for the prediction of RNA secondary structures with pseudoknots. This approach allows us to combine two thermodynamic models into a single bi-objective integer program (BOIP), from which we can get the set of optimal secondary structures having the best trade-off between the two criteria. Note that a method to find bi-objective optimal solutions for the RNA folding problem, combining also two thermodynamic models, namely the MEA and the MFE models, was also developed [14]. This method defines a binary Pareto product operator using algebraic dynamic programming and studies different implementations of this operator. The authors showed that this combination generates Pareto sets with some diversified structures with their variations. As stated before, sub-optimal solutions are equally of great interest from a biological point of view. We therefore propose an algorithm to retrieve the *k*-best (sub-)optimal solutions for any BOIP and apply it to our specific issue. In this work, we consider a first model based on the MEA model proposed in [10], to which we will refer as Mod1. A second model, based on the MFE model proposed in [6], will be refered as Mod2. We have thus performed the following steps:

- We developed an original generic algorithm, that allows to return several optimal and several sub-optimal solutions for any BOIP.
- We combined the two thermodynamic models Mod1 and Mod2 for prediction of RNA secondary structure with pseudoknots into one BOIP.
- We implemented this BOIP with our generic algorithm to predict several optimal and several sub-optimal RNA secondary structures. The tool is called BiokoP (Bi-objective programming pseudoknot Prediction) and is available on our EvryRNA platform.

We evaluated our algorithm on a dataset of 198 pseudoknotted RNA sequences from PseudoBase++ [20]. The first observation is that the real structure is often given by a sub-optimal solution, which confirms the need of returning sub-optimal solutions. BiokoP was then compared with other tools proposing several solutions for pseudoknotted RNA secondary structure prediction. To our knowledge, only two tools are available in the literature, namely pKiss [4] and McGenus [5]. BiokoP was also compared to IPknot [10], in the case where one solution is returned. Considering the dataset of pseudoknotted secondary structures, BiokoP gives better F_1_-scores than the other tools. The results in function of the type of pseudoknots show that BiokoP gives homogeneous results regardless of the pseudoknot type. Indeed, the F_1_-scores are always higher than 70% for any number of solutions returned, contrary to those of pKiss and McGenus. The results also show that BiokoP is more likely to return the best structure (according to the F_1_-score) among the optimal solutions than the other tools. We also experimented BiokoP on a dataset of pseudoknot-free RNA sequences from RNA STRAND [21]. We compared BiokoP on this dataset with the other tools and with RNAsubopt [22]. RNAsubopt is able to predict pseudoknot-free structures and sub-optimal solutions. The results show that BiokoP is able to predict pseudoknot-free secondary structures with F_1_-scores close to those of RNAsubopt and better than those of pKiss and McGenus.

The paper is organized as follows: in the “Methods” section, we start by giving some fundamental definitions in multi-objective optimization. We present our algorithm, which aims to compute several solutions (optimal and sub-optimal), for any BOIP. Then, we present how we combined the two models Mod1 and Mod2 into a single BOIP to predict RNA secondary structures with pseudoknots. The “Results” section is devoted to the experimental evaluation of our method. Finally, we discuss about our results in the “Discussion” section and we conclude and give some perspectives in the “Conclusion” section.

## Methods

Our work is based on integer programming which consists in optimizing an objective function according to linear constraints over a set of integer decision variables [23]. It allows to model very different problems. Integer programming is usually used to obtain an optimal solution, but here, the purpose is to obtain also several sub-optimal solutions.

We are interested in optimizing several objective functions, corresponding here to different models for RNA secondary structure prediction. We thus have a bi-objective integer program, and the set of optimal solutions is called the *Pareto set*. As said before, regarding our biological context, we are interested in finding optimal and sub-optimal solutions. In a multi-criteria setting, it means to compute sub-optimal Pareto sets, namely the *k*-best Pareto sets for *k*≥1. Hence, we present a new method to generate those sets for a generic bi-objective integer program (BOIP). We would like to stress out that this is a totally new problem to our knowledge, this should not be confused with the traditional problem of finding approximate Pareto sets. Indeed, in the latter approach, one wants to find an approximation of the exact Pareto set, whereas in our method we find the exact (sub-)optimal Pareto sets.

### The bi-objective integer programming

**Fig. 1:**
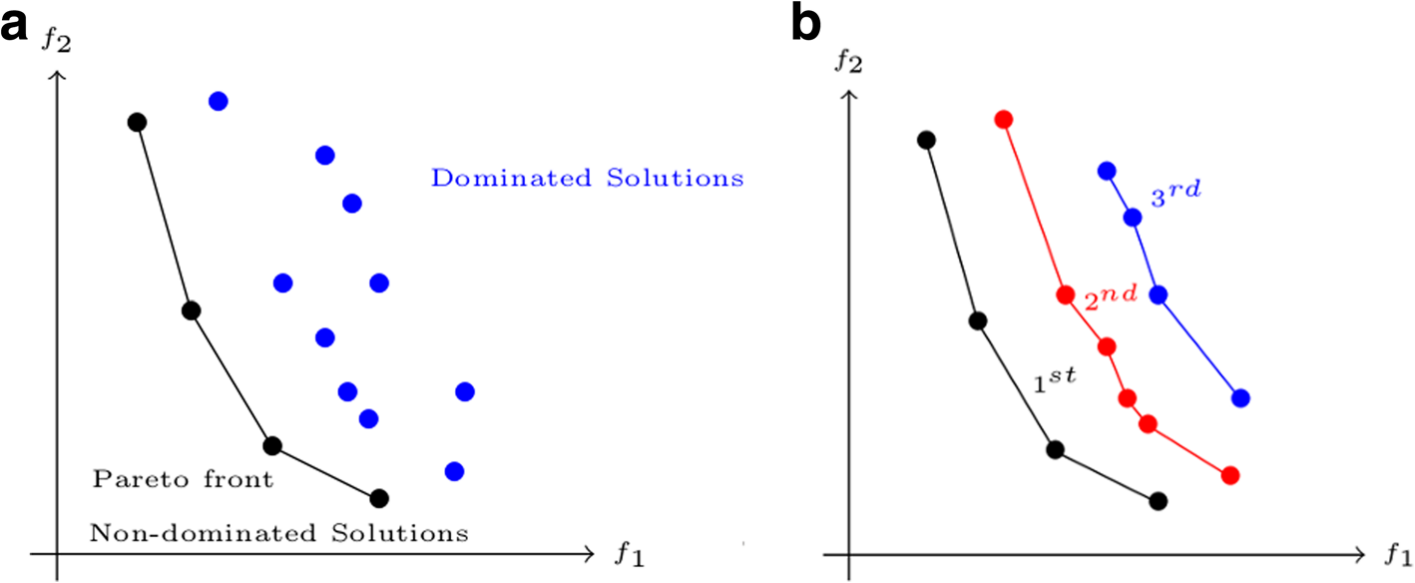
Pareto front, Pareto set and *k*-best Pareto set according to two objectives to minimized. **a** The set of non-dominated solutions is the Pareto set, and their corresponding values according to the two criteria form the Pareto front. **b** Example of *k*-best Pareto sets with *k*=1,2,3

A multi-objective integer program (IP) is an IP with more than one objective function. In the sequel, we consider the case where there are only two objective functions, denoted by *f*_1_ and *f*_2_, and one wants to minimize them. In that case we say that we have a BOIP. Given a BOIP, we denote by $\mathcal X$ its set of feasible solutions, i.e., the set of solutions satisfying all constraints. Let *x* and *x*^′^ in $\mathcal X$ be two solutions. We say that *x* dominates *x*^′^, denoted by *x*≻*x*^′^, if and only if *f*_1_(*x*)≤*f*_1_(*x*^′^) and *f*_2_(*x*)≤*f*_2_(*x*^′^), where at least one inequality is strict. Since, in general, there does not exist a solution dominating all other solutions, we are looking for a trade-off. A solution $x\in \mathcal X$ is Pareto efficient if and only if there does not exist a solution $x'\in \mathcal X$ such that *x*^′^≻*x*. The *Pareto set* is ${\mathcal P} := \{x\in {\mathcal X}: x\ \text {is Pareto efficient}\}$. It is the set of solutions which are not dominated by other solutions. The *Pareto front* is ${\mathcal F} := \left \{\left (f_{1}(x),f_{2}(x)\right) : x\in {\mathcal P} \right \}$. Figure 1a illustrates those definitions.

Many methods exist to solve multi-objective combinatorial optimization problems and BOIP. There are methods for finding the exact Pareto front [24–28] or an approximation of it [29, 30]. A first difference of our approach with the majority of the above works is that we are rather interested in finding the Pareto set instead of the Pareto front, and in case there are several solutions with the same values for each objective function, we want to find them all. Another more fundamental difference is that we are also interested in computing sub-optimal Pareto sets, namely the *k*-best Pareto sets with *k*≥1. For example, the second best Pareto set corresponds to the best trade-off when the solutions belonging to the first Pareto set have been removed. In other words, when the first Pareto set is removed, the remaining non-dominated solutions form the 2-best Pareto set. Figure 1b shows several *k*-best Pareto sets.

### Algorithm for finding the *k*-best Pareto sets

In this section, we present an original generic algorithm we developed to compute the *k*-best Pareto sets for any BOIP:

min *f*_1_(*x*)

min *f*_2_(*x*)

subject to:

$$\begin{array}{c} g_{k}(x) \leq 0 \qquad k=1,\dots,m\\ x=(x_{1},x_{2},\dots,x_{n})\\ x_{i} \in {\mathbb Z} \qquad 1 \leq i \leq n \end{array} $$

The constraints are described here as linear functions *g*_*k*_ of *x*.

For the clarity of the presentation let us assume first that all the variables in the BOIP are binary ones. In that case, given a set *F* of forbidden solutions, we denote by *P*_1_(*λ*_*min*_,*λ*_*max*_,*F*) the following IP:

min *f*_1_(*x*)

subject to:

$$\begin{array}{c} f_{2}(x) \geq \lambda_{min}\\ f_{2}(x) \leq \lambda_{max}\\ \text{DIFF}(s) \text{ for } s\in F\\ g_{k}(x) \leq 0 \qquad k=1,\dots,m\\ x=(x_{1},x_{2},\dots,x_{n})\\ x_{i} \in {\mathbb Z} \qquad 1 \leq i \leq n \end{array} $$

In this IP the first objective function *f*_1_ to be minimized stays the same. The second objective function *f*_2_ is introduced by two constraints which will maintain its value between *λ*_*min*_ and *λ*_*max*_.

For each solution *s* in *F*, a constraint DIFF (*s*), also present in [31], is added. This constraint forbids to find the solution in *F* again. The constraint is defined in the following way. Let assume we have found a solution $x^{*}=\left (x^{*}_{1},x^{*}_{2},\dots,x^{*}_{n}\right) \in F$ of a binary IP. Let define $B:=\left \{ i | x^{*}_{i}=1\right \}$ and $N :=\left \{ i | x^{*}_{i}=0\right \}$. The DIFF (*x*^∗^) constraint is: $\sum _{i\in B}(1-x_{i}) + \sum _{i\in N}{x_{i}} \geq 1$. This constraint ensures that the (Hamming) distance between any feasible solution *s* and the solution *x*^∗^ is at least one. Therefore, there must be at least one variable *x*_*i*_ which takes a different value from $x^{*}_{i}$.

For the more general case, i.e. for BOIP with integer decision variables, this time, several binary and continuous variables together with several constraints must be added to the IP, leading to a mixed linear program [32]. For each solution $x^{*} = \left (x^{*}_{1},x^{*}_{2},\dots,x^{*}_{n}\right)\in F$, we create the *n* binary variables *α*_*i*_∈{0,1} for 1≤*i*≤*n*, and the *n*+1 continuous variables, *W*_*i*_≥0, (1≤*i*≤*n*) and 0≤*θ*≤1, together with the following constraints (*M* being a large constant):

$$\begin{cases} 0 \leq W_{i} - x_{i} + x^{*}_{i} \leq M(1-\alpha_{i}), & 1 \leq i \leq n \\ 0 \leq W_{i} - x^{*}_{i} + x_{i} \leq M\alpha_{i}, & 1 \leq i \leq n \\ \sum_{i=1}^{n} W_{i} + \theta \geq 1\\ \end{cases} $$

Of course, these modifications do not change the main algorithm, their aim is to forbid the solutions in *F*. In the following, we denote again by *P*_1_(*λ*_*min*_,*λ*_*max*_,*F*) the resulting mixed linear program.

We denote by *P*_2_ the following IP:

max *f*_2_(*x*)

subject to:

$$\begin{array}{c} g_{k}(x) \leq 0 \qquad k=1,\dots,m\\ x=(x_{1},x_{2},\dots,x_{n})\\ x_{i} \in {\mathbb Z} \qquad 1 \leq i \leq n \end{array} $$

The general idea of our algorithm is to recursively perform a dichotomic search in the areas above and below each new solution found. We denote by *nb* the number of Pareto sets seeked. At the end of the algorithm, the set $\mathcal R$ will contain all the solutions belonging to the *k*-th Pareto sets, for 1≤*k*≤*nb*. For each solution *s* found during the execution of the algorithm, we have a label, denoted by *l*(*s*), indicating the index of the set this solution belongs to, i.e., *l*(*s*)=*k* iff the solution *s* belongs to the *k*-th Pareto set.

Our algorithm, called *FindKParetoSets* works as follows. First, we find a (leftmost) solution *L*, minimizing the *f*_1_ criterion. We set its label to 1, *l*(*L*):=1, and this solution is added to the set $\mathcal R$. Notice that since there can exist several solutions minimizing *f*_1_ with different *f*_2_ values, this solution does not necessarily belong to the first Pareto set. In that case, its correct label will be set during the remaining execution of the algorithm. Then, we compute the solution *U* maximizing the *f*_2_ criterion. An *f*_1_ value of a solution *s* is noted as *s*_1_, and in the same manner, *s*_2_ defines the *f*_2_ value. In the following, *U*_2_ will serve as an upper bound for the recursive search. Finally the *localPareto()* procedure is called and performs the recursive search, first below *L*, between −*∞* and *L*_2_−*ε* according to the *f*_2_ criterion, and then above *L*, between *L*_2_ and *U*_2_. Here *ε* is a very small constant such that for any pair of solutions *s*,*s*^′^ one has either *f*_2_(*s*)=*f*_2_(*s*^′^) or |*f*_2_(*s*)−*f*_2_(*s*^′^)|>*ε*.



The *localPareto()* procedure is described below. Each search, corresponding to the computation of a portion of a Pareto set, is done between two values, denoted by *λ*_*min*_ and *λ*_*max*_, that are taken as two arguments. The set *F* represents a set of solutions previously found between *λ*_*min*_ and *λ*_*max*_, that we could find again by solving *P*_1_. To avoid it, the solutions of *F* are forbidden as explained before. If the IP *P*_1_(*λ*_*min*_,*λ*_*max*_,*F*) has a solution *s* (lines ??-??), by default its label is set to 1 (line ??). Then, the label of *s* must be computed according to lines ??-??. If the label is inferior or equal to *nb*+1, the solution *s* is added to ${\mathcal R}$. If necessary, the labels of some previously found solutions of $\mathcal R$ are updated (lines ?? to ??). Finally, the *localPareto()* procedure is called to search below *s* (between *λ*_*min*_ and *s*_2_−*ε*) and above *s* (between *s*_2_ and *λ*_*max*_) if the label is inferior to *nb*.



**Fig. 2:**
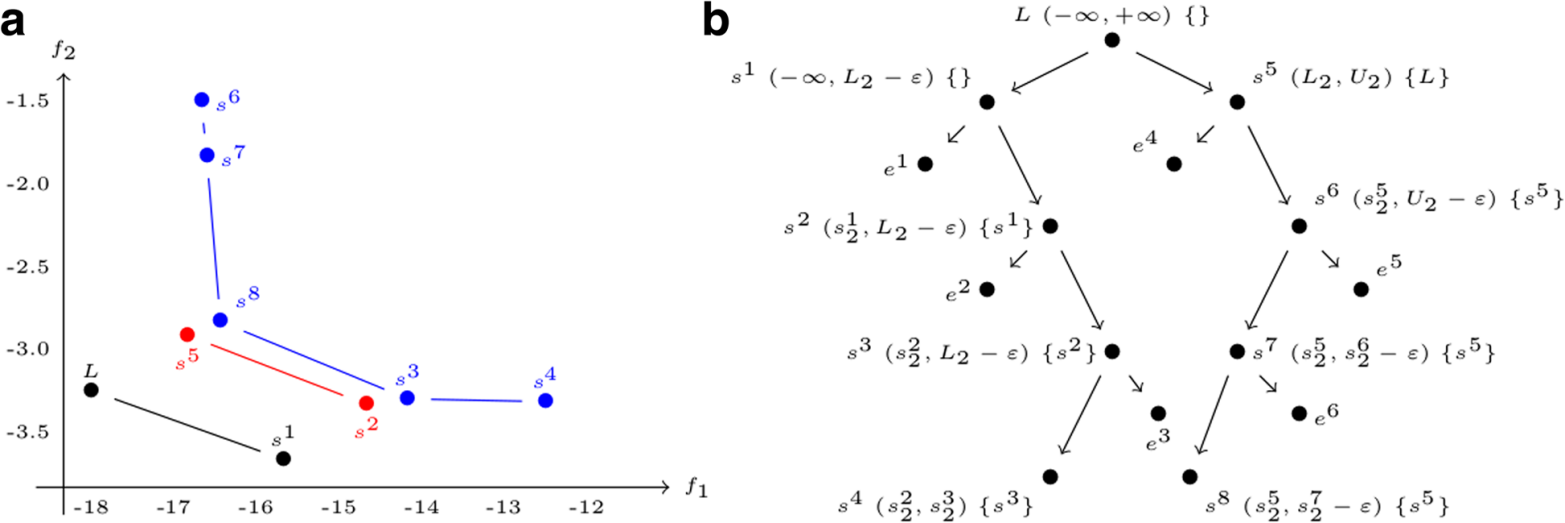
Example results of the *FindKBestParetoSets* algorithm. **a** Results of the determination of three Pareto sets with the algorithm for the PKB101 RNA from satellite tobacco mosaic virus. For each solution is displayed the identifier *s*^*i*^. **b** Recursive calls of the algorithm. For each call is displayed the identifier of the current solution *s*^*i*^, the search space (*λ*_*min*_, *λ*_*max*_) and the set *F*. A *e* represents no solution or a solution whose the label is superior to *nb* or *nb*+1. The left branches are the searches below the current solution *s* and the right branches are the searches above the current solution *s*

**Example** We show an example of an execution of the algorithm *FindKParetoSets* to find three Pareto sets. We solve the BOIP presented in the following section, with the PKB101 RNA from the satellite tobacco mosaic virus. Figure 2 shows the three Pareto sets obtained and summarizes the recursive search.

The first step of our algorithm is to find the solution denoted *L*, by solving the BOIP (line ??), and add it to the set ${\mathcal R}$ (line ??). Then a maximum threshold *U*_2_ is found by solving *P*_2_ (line ??) to search above the first solution *L*. A search below the solution *L* is done (line ??) and the solution *s*^1^ is found. In the *localPareto()* procedure, the solution *s*^1^ obtains the label of the previous solution *L*. A search below *s*^1^ is done, but no solution is found. The search above *s*^1^ is done and *s*^2^ is found. The recursive search continues until no additional solution is found.

### Bi-objective integer programming for predicting RNA secondary structures with pseudoknots

In this paper, we propose a method for predicting RNA secondary structures with pseudoknots using the algorithm presented based on a BOIP. Our method allows to return several optimal and several sub-optimal solutions, optimizing two objectives related to an MEA model and an MFE model. The MEA model, to which we will refer as Mod1, is based on the model proposed in [10] and uses the Dirks and Pierce set of thermodynamic parameters [8]. The MFE model, to which we will refer as Mod2, is based on the model proposed in [6]. Mod1 and Mod2 can describe all kinds of pseudoknots. In the following, we present first how an RNA structure with pseudoknots can be modeled. Then we describe how we combine Mod1 and Mod2 into one BOIP.

#### Modeling RNA secondary structures with pseudoknots

In Mod1 and Mod2, the RNA secondary structures are modeled in the following way. An RNA sequence *s* is composed of *n* nucleotides or bases which can be A, U, G or C. Each base can be paired according to the Watson-Crick (A-U and G-C) or the Wobble (G-U) pairings. To take into account the pseudoknots, it is assumed that a secondary structure can be decomposed into *m* pseudoknot-free substructures *y*^1^, *y*^2^,…, *y*^*m*^, called levels. The levels are disjoint sets meaning that a base pair belongs to exactly one level. From experimental data, it is generally assumed that two levels are sufficient to describe most known RNA structures. Then, in the following, *m*=2.

A base pair between the bases *i* and *j* in level *p* is represented by a binary variable $y_{ij}^{p}$ equal to 1, with *i*=1,…,*n* and *j*=*i*+1,…,*n*. If there is no base pair between *i* and *j*, $y_{ij}^{p}$ is equal to zero.

The possible types of base pairs correspond to integer values 1,…,6: A-U has the value 1, C-G the value 2, G-C the value 3, G-U the value 4, U-G the value 5 and U-A the value 6.

The possible stacks of two base pairs (*i*,*j*) and (*i*−1,*j*+1) in level *p* are defined with binary variables $x_{ij}^{klp}$, with *k* and *l* representing the possible types of base pairs. If $x_{ij}^{klp}$ is equal to 1, then the bases *i* and *j*, and the bases *i*+1 and *j*−1 are paired, and in the case where $x_{ij}^{klp}$ is equal to zero, there is either one base pair or no base pair at all.

#### Predicting RNA secondary structures with pseudoknots by combining two models

In the BOIP, we combine Mod1 and Mod2. The objective of Mod1 is to find the MEA structure with none pseudoknot or with one or several pseudoknots of any type.

The MEA structure is found by the computation of base pair probabilities with the Dirks and Pierce model [8]. We set as *f*_1_ the approximation of the expected accuracy:

1 $$ f_{1}(y) = \sum_{1\leq p \leq m}{\beta^{p}} \sum_{i < j s.t. p_{ij} > \theta^{p}}{p_{ij}y_{ij}^{\, p}}  $$

where *β*^*p*^ are constants for each level *p*, fixed to *β*^*p*^=1/*m*, *p*_*ij*_ are the base pair probabilities computed with the Dirks and Pierce model and *θ*^*p*^ is a threshold aiming to ignore the lower base pair probabilities.

The objective of Mod2 is to seek the MFE structure. The MFE function consists in the sum of the energies of each stack $x_{ij}^{klp}$ of two base pairs:

2 $$ f_{2}(x) = \sum_{p=0}^{m}\sum_{i=1}^{n}\sum_{j=1}^{n}\sum_{k=1}^{6}\sum_{l=1}^{6} e_{kl}x_{ij}^{klp}  $$

with *e*_*kl*_ the energy given in [6], depending on the types *k* and *l* of the two base pairs.

For the need of the algorithm, the sign of the function *f*_1_(*y*) is changed to have two objective functions to minimize.

**Fig. 3:**
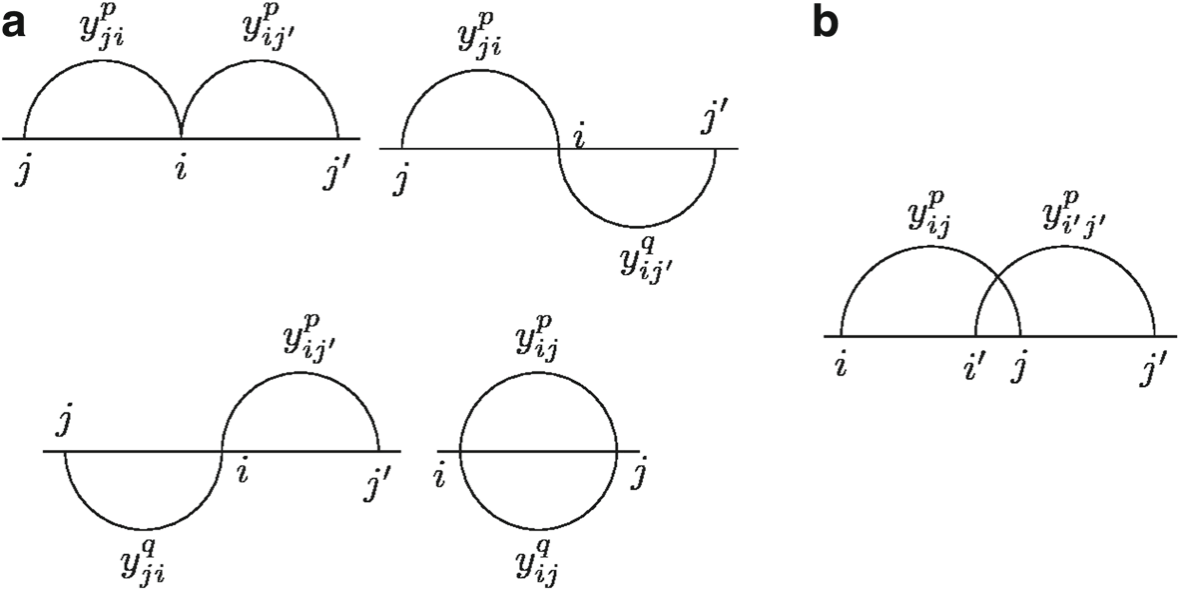
Different cases of forbidden base pairs in RNA secondary structures with pseudoknots. **a** The base *i* of level *p* cannot be paired with several bases at the same time, from the same or different level; and the base pair between the bases *i* and *j* cannot exist on two different levels *p* and *q* at the same time. **b** Two base pairs *ij* and *i*^′^*j*^′^ forming a pseudoknot cannot exist at the same level *p*

The constraints of the BOIP enforce that any feasible solution corresponds to a feasible folding configuration of a secondary structure of RNA. They define basic rules (Fig. 3) such as making impossible for a base *i* to be paired with several bases, forbidding the presence of pseudoknots on the same level and forbidding isolated base pairs. Also, adding pseudoknots in the structure is penalized since they are rare, according to the known structures. The DIFF constraints will be added for any solution in F. This constraint adapted to our BOIP is:

3 $$ \begin{aligned} \sum_{p=1}^{m}\sum_{ij \in B^{p}} {y_{ij}^{\, p}} & - \sum_{p=1}^{m}\sum_{ij \in N^{p}}{y_{ij}^{p}}\\ & \leq \sum_{p=1}^{m}{| B^{p} |} - 1 \quad (1 \leq \forall p\leq m, \forall s \in F) \end{aligned}  $$

with $B^{p}=\left \{ ij | y_{ij}^{*p}=1\right \}$ and $N^{p} =\left \{ ij | y_{ij}^{*p}=0\right \}$.

In our BOIP, the pseudoknot levels can be inverted, causing the generation of different solutions (that have not necessarily the same objective values) corresponding to the same structure. To avoid this redundancy, the following constraint is added:

4 $$ \sum_{ij \in B^{2}} {y_{ij}^{1}} + \sum_{ij \in B^{1}} {y_{ij}^{2}} - \sum_{ij \in N^{2}}{y_{ij}^{1}} - \sum_{ij \in N^{1}}{y_{ij}^{2}} \leq | B^{1} | + | B^{2} | - 1  $$

This constraint corresponds to the previous constraint but the levels of the sets *B* and *N* are inverted. Then, the base pairs of the level 1 are forbidden in level 2 and vice versa.

## Results

The BOIP presented for predicting RNA secondary structures with pseudoknots is implemented using the CPLEX Optimizer V12.6.3 solver [33]. Our algorithm is implemented with *ε*=0.001 and *m*=2. The obtained tool, called BiokoP, is available on our EvryRNA platform.

In the following, we first present the datasets we use for the evaluation of BiokoP, then the experiments showing the distribution of real structures found over the generated solutions. The next section is devoted to a statistical analysis of structures predicted by BiokoP and by other tools from the literature. We end by giving some information on the execution time of BiokoP.

### Datasets

We evaluate our approach on a dataset of pseudoknotted RNAs we built from the PseudoBase ++ database [20]. This dataset gathers 198 sequences whose lengths range from 21 to 128 nucleotides.

**Fig. 4:**
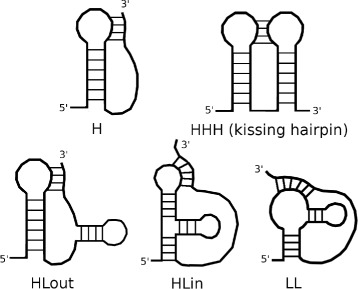
RNA pseudoknot types. RNA pseudoknot types from Pseudobase++ [20] classification

PseudoBase++ classifies the sequences by the pseudoknot types. We recovered five types of pseudoknots: H (H-type), HHH (kissing hairpin), HLout, HLin and LL. The types, described in Fig. 4, are defined in function of the topology of the pseudoknot. In our dataset, there are 154 pseudoknotted RNAs of type H, 3 of type HHH, 26 of type HLout, 4 of type HLin and 11 of type LL. All the RNAs of type H come from the dataset of 168 sequences built by Huang et al. [34] from PseudoBase ++. This dataset excludes redundant sequences. The remaining RNAs were recovered on the database by requests according to the type of pseudoknots.

We also built a second dataset of pseudoknot-free RNAs from the RNA STRAND database [21]. It gathers 145 non-redundant sequences whose lengths range from 10 to 97 nucleotides.

These datasets are available on the EvryRNA platform.

### Distribution of real structures over the returned solutions

In this section we study the ability of BiokoP to find the real structures. The purpose is to analyze where the real structures are found, over the Pareto sets or in function of the number of solutions returned. This section is also devoted to a comparison between BiokoP, Mod1 and Mod2 in order to determine the contribution of BiokoP.

#### Distribution of real structures over the Pareto sets

We study the distribution of real structures returned by BiokoP on our dataset of pseudoknotted RNAs over the Pareto sets. The real structure is the structure that corresponds exactly to the referenced structure for a given RNA.

To study the distribution of real structures, as the number of solutions of a Pareto set can not be predicted, note that in order to have 30 solutions per RNA, the mean number of Pareto sets to compute is 5.2. The distribution of real structures found are displayed in Table 1. Around the half of real structures found are in the first Pareto set (45 over 83). These structures are the optimal ones, showing the relevance of combining these MEA and MFE models. The real structures corresponding to sub-optimal solutions are distributed in the first sub-optimal Pareto sets, mainly the second (15) and the third (13). The remaining solutions are scattered in the remaining Pareto sets. The position of these sub-optimal solutions supports with the fact that the real structure is often a sub-optimal solution. This suggests that the sub-optimal solutions returned by BiokoP are diversified and that our approach finding the *k*-best Pareto sets allows to find pertinent sub-optimal solutions. Finally, it appears that the first Pareto sets are more useful for this combination of models than the last Pareto sets which do not guarantee to find the real structure. Indeed, the quality of solutions decreases when the number of computed Pareto sets increases. Hence, we recommend to the users to compute three Pareto sets in mean to obtain a relevant set of solutions.

**Table 1:** Distribution of real structures found by BiokoP in function of Pareto sets

*k*-best Pareto set, *k*=	1	2	3	4	5	6	7	8	9	10	Total
Number of real structures	45	15	13	7	1	0	0	0	1	1	83

#### Distribution of real structures in function of the number of solutions returned

**Fig. 5:**
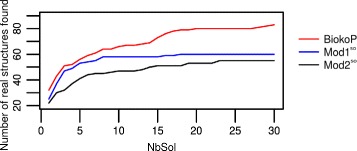
Distribution of real structures found by BiokoP, Mod1^so^ and Mod2^so^ on the dataset of pseudoknotted RNAs in function of the number of solutions returned (NbSol)

This section is devoted to the distribution of real structures found by BiokoP in function of the number of solutions returned on our dataset of pseudoknotted RNAs, and to the comparison with Mod1 and Mod2, in order to show the pertinence of combining these two models on one hand, and to return several solutions on another hand. We extended Mod1 and Mod2 so that they return the *k*-best solutions, using the constraint [31] presented in the “Algorithm for finding the *k*-best Pareto sets” section. We refer to these extensions as Mod1^so^ and Mod2^so^ (*so* stands for sub-optimal). The results are reported in Fig. 5.

BiokoP is made to return sets of solutions and all the solutions belonging to one Pareto set are not comparable. Then, this experiment requires to rank the solutions of the Pareto sets returned by BiokoP in order to compare the solutions one against the others. The solutions of each Pareto set are ranked in the following manner: the solutions optimizing equally the two objectives, i.e., the solutions closer to the diagonal, are better ranked.

The results on the dataset of pseudoknotted RNA show that, as expected, BiokoP predicts more real structures than Mod1 and Mod2 (corresponding respectively to Mod1^so^ and Mod2^so^ for one solution returned). Indeed, BiokoP, Mod1 and Mod2 return the real structure for respectively 32, 25 and 23 RNAs. We observe that, in these sets of real structures returned by Mod1 and Mod2, 12 RNAs are identical. Those RNAs also show up in the set of real structures returned by BiokoP. In the remaining real structures found by BiokoP, 6 are neither found by Mod1 nor by Mod2. This shows clearly the pertinence of combining Mod1 and Mod2. Besides, we note that BiokoP finds all the real structures found by Mod1. Some real structures found by Mod2 are not found by BiokoP when one solution is returned but they are all found by BiokoP in the first Pareto set. The real structures found by Mod1 and Mod2 are all returned by BiokoP as optimal solutions, showing that our algorithm succeeds to take benefit from both models.

The more there are solutions, the more BiokoP is likely to find the real structure, and with a fast increase in probability. We observe that after about 20 solutions returned (for about 2 or 3 Pareto sets), the number of real structures found seems to be stable, which supports the results of the previous section. In case of Mod1^so^ and Mod2^so^, the number of real structures found quickly reaches a plateau amounting to 7- 8 solutions returned. This is due to the lack of diversity of the sub-optimal solutions. Indeed, the sub-optimal solutions are essentially similar to the optimal one: they are derived from the optimal solution by removing only very few base pairs. When the optimal solution is close to the real structure, the real structure can be found quickly as a sub-optimal solution, explaining the increase of the curve for a small number of returned solutions.

Finally, this experiment shows that the optimal and sub-optimal solutions returned by BiokoP are more likely to contain the real structure compared to those of Mod1^so^ and Mod2^so^.

### Comparison of BiokoP with the literature

#### Considered software

To evaluate the performances of BiokoP, we compare it with other methods predicting pseudoknotted RNA secondary structures that are able to return several solutions. To our knowledge, only two methods are available in the literature, namely pKiss [4] and McGenus [5]. The principle of pKiss is to decompose the RNA sequence into every possible sub-words and to compute the MFE secondary structure of the decompositions. To reduce the search space, pKiss is based on the canonical rules which reduce the number of possible predicted pseudoknots (only certain canonical and kissing pseudoknots) and the redundancy thanks to a non-ambiguous dynamic programming algorithm. McGenus is based on a Monte Carlo algorithm which search for a minimum score which includes the energy and the genus of the secondary structure. The genus expresses the complexity of a pseudoknot. McGenus performs a stochastic search that allows to find various types of pseudoknots.

We also compare BiokoP with IPknot [10] and RNAsubopt from the ViennaRNA package [22]. RNAsubopt predicts pseudoknot-free RNA secondary structures using an MFE algorithm to compute all the sub-optimal structures in an energy range.

For the evaluation, we consider the first solution returned by IPknot and the 30 first solutions returned by BiokoP, pKiss, McGenus and RNAsubopt. IPknot (version 0.0.4) was executed with the Dirks and Pierce set of thermodynamic parameters and with the options -g 2 and -g 4. pKiss (version 2.2.12) was executed with the default parameters. We used the option -relativeDeviation to obtain up to 30 solutions for each RNA. McGenus (version 7.0) was also executed with the default parameters, with the option -nsuboptimal to obtain 30 solutions. We executed RNAsubopt (version 2.3.3) with the option -e to obtain 30 solutions and with the option -s to sort the solutions by energy.

For pKiss, McGenus and RNAsubopt, the solutions are ranked in the returned order, i.e., in the ascending order of energies. For BiokoP, as the solutions belonging to the same Pareto set are returned in an arbitrary order and are not comparable, we adopt the same ranking as in the previous section. We consider that the best solutions are the ones that optimize equally the two objectives, and are therefore closer to the diagonal.

#### Statistics used

To evaluate the quality of a predicted structure, the statistics usually used are the sensitivity, the positive predictive value (PPV) and the F_1_-score. The sensitivity measures the ability of finding positive base pairs, while the PPV measures the ability of not finding false positive base pairs. The F_1_-score is the harmonic mean between the sensitivity and the PPV. The three measures are calculated as follows:

$$\begin{aligned} Sensitivity &=\frac{TP}{TP+FN},\ \ {PPV}=\frac{TP}{TP+FP}, \\ \text{F}_{1}\text{-score} &=2 \times \frac{Sensitivity \times {PPV}}{Sensitivity + {PPV}}, \end{aligned} $$

where *TP* is the number of true positive base pairs, *FN* is the number of false negative base pairs, *FP* is the number of false positive base pairs, and *TN* is the number of true negative base pairs. These statistics allow to measure the quality of one solution regarding a structure of reference. In our case, we study methods returning several solutions; therefore, these statistics should be adapted to be able to measure the quality of a set of *n* solutions regarding a structure of reference. Here we propose to calculate these measures as follows:

$$M=\frac{\sum_{i=1}^{n} M(s_{i}) \times (n-i+1)}{n} $$

where *M*, a measure corresponding for instance to the F_1_-score of a set of solutions, is calculated in function of the measure *M*(*s*_*i*_) corresponding to the F_1_-score of a solution *s*_*i*_, weighted by the rank *i* of the solution. Of course, the more the rank of a solution is low, the more the solution is important, since the corresponding criteria are optimized.

#### Overall results

In this section are presented the results obtained on the dataset of 198 pseudoknotted RNAs. Table 2 reports the weighted means of sensitivities and PPVs in function of the number of solutions returned for BiokoP, pKiss and McGenus. We observe that BiokoP has better sensitivities than pKiss and McGenus and that, when the number of returned solutions increases, the gap between the sensitivity of BiokoP and the one of the other tools increases. Regarding the weighted means of PPVs, we observe that BiokoP outperforms McGenus.

**Table 2:** Sensitivity and PPV results for BiokoP, pKiss and McGenus on pseudoknotted RNAs

NbSol	BiokoP		pKiss		McGenus		BiokoP		pKiss		McGenus	
Sensitivity s.d.					PPV s.d.							
1	*80.6*	22.3	79.5	24.2	73.4	26.6	75.0	25.5	*75.1*	26.6	74.1	28.6
2	*80.6*	22.3	77.6	23.5	67.1	26.7	73.2	25.5	*74.8*	26.2	69.7	31.6
3	*80.1*	22.4	75.8	23.6	63.8	30.5	71.6	25.7	*74.2*	26.4	67.3	32.7
4	*79.5*	22.8	74.4	23.8	62.2	30.8	70.5	26.0	*73.5*	26.6	66.2	33.3
5	*79.0*	23.1	73.1	24.0	61.0	31.0	69.7	26.2	*72.9*	26.8	65.5	33.6
10	*77.0*	23.6	68.8	24.1	57.4	31.9	67.4	26.4	*70.9*	27.1	62.6	35.3
15	*75.8*	23.6	65.7	24.5	55.7	32.6	66.4	26.4	*69.3*	27.6	61.0	36.1
20	*75.1*	23.6	63.2	25.0	54.2	33.1	65.9	26.4	*67.9*	28.1	59.4	36.8
25	*74.5*	23.5	61.2	25.3	53.3	33.4	65.5	26.3	*66.7*	28.5	58.4	37.2
30	*73.8*	23.5	59.5	25.5	53.3	33.4	65.0	26.3	*65.2*	28.9	58.5	37.2

Weighted means of sensitivities and PPVs with standard deviations (s.d.) for BiokoP, pKiss and McGenus according to the number of solutions (NbSol), on a set of 198 pseudoknotted RNAs

A value in italic means this value is the best among the three tools

**Fig. 6:**
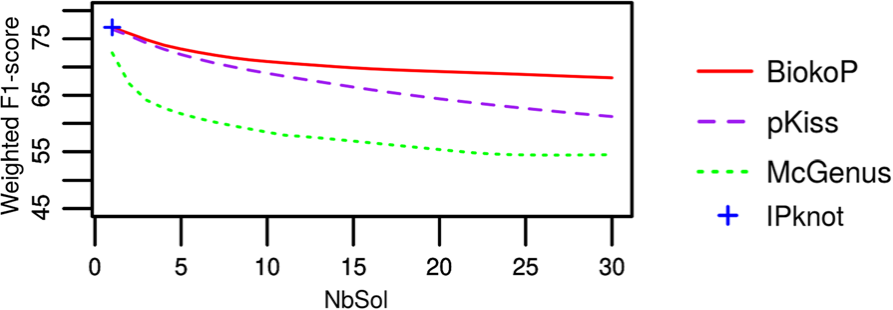
F_1_-score results on pseudoknotted RNAs. Weighted means of F_1_-scores of the structures predicted with BiokoP, pKiss, McGenus and IPknot, in function of the number of solutions (NbSol) on a dataset of 198 pseudoknotted RNAs

In Fig. 6 we present the weighted means of F_1_-scores obtained by each tool, in function of the number of solutions returned. BiokoP has higher F_1_-scores than pKiss and McGenus. The F_1_-scores of BiokoP are quite stable. There is only a decrease of 10 points going from 1 to 30 returned solutions, whereas there is a decrease of 15 and 18 points for pKiss and McGenus. This suggests that the quality of predicted structures of BiokoP, unlike pKiss and McGenus, is stable when the quantity of returned solutions increases.

For one solution returned, BiokoP gives similar results to IPknot (IPknot gives a mean sensitivity of 80.6%, a mean PPV of 75.1% and a mean F_1_-score of 77,0%).

#### Results over optimal solutions

**Fig. 7:**
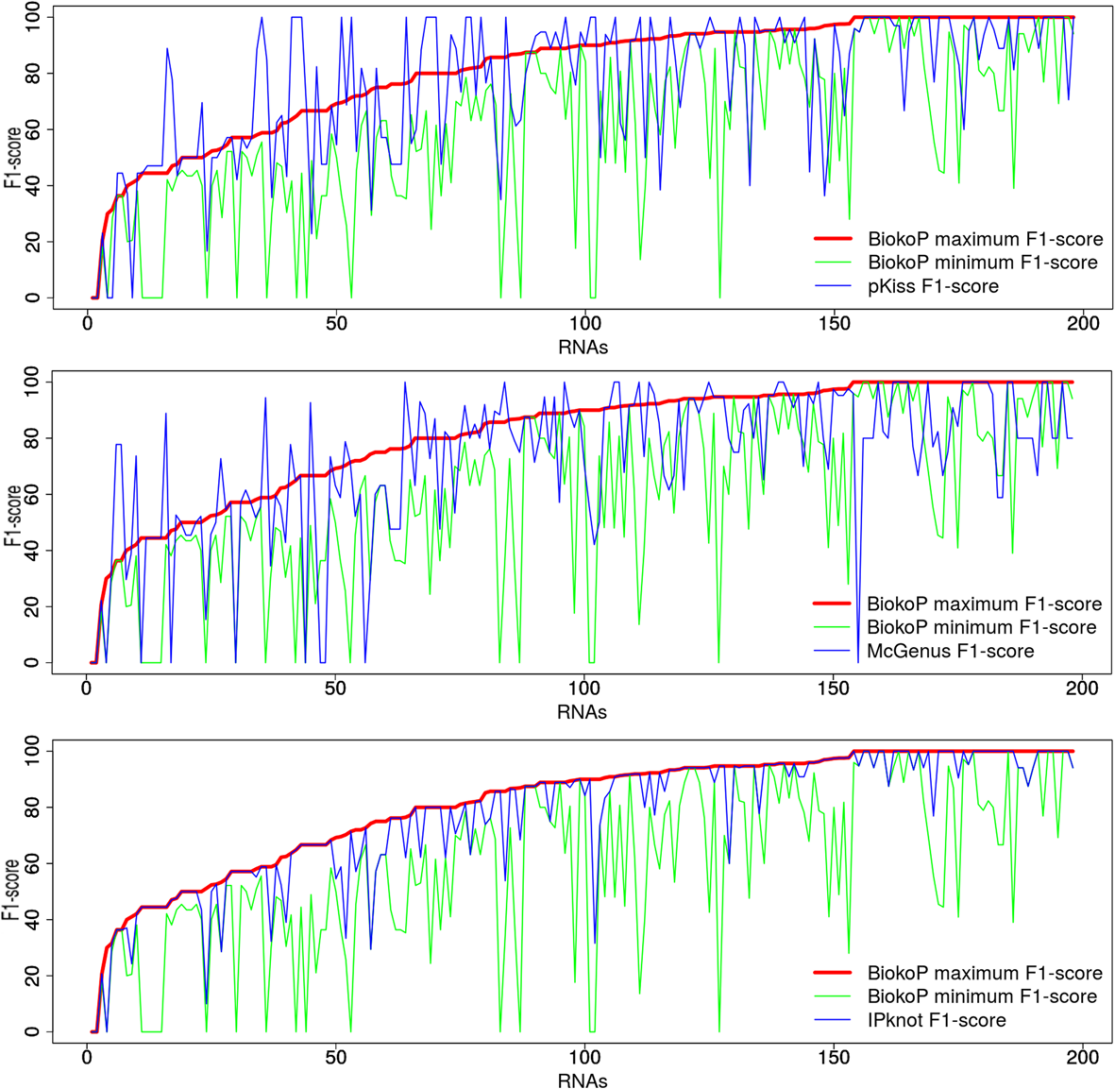
F_1_-score results of the optimal solutions for each pseudoknotted RNA. Maximum and minimum F_1_-scores of the optimal solutions returned by BiokoP, and F_1_-score of the optimal solution returned by pKiss (top), McGenus (middle) and IPknot (bottom) compared among 198 pseudoknotted RNAs. The RNAs are sorted according to the ascending order of the maximum F_1_-score of BiokoP

The purpose of this section is to complete and to precise the results given by the previous statistics. It is not obvious to compare the optimal solution returned either by pKiss, McGenus or IPknot with only one solution obtained by an arbitrary ranking of the solutions of the optimal Pareto set given by BiokoP. Indeed, the solutions of a Pareto set are not comparable. We thus focus here on the comparison of the first solutions returned, i.e. the optimal solutions of BiokoP (the first Pareto set) and the optimal (one) solution returned by the other tools. Figure 7 reports the F_1_-score results for the optimal solutions of BiokoP versus pKiss, McGenus and IPknot, for each RNA of the dataset of pseudoknotted RNAs. The RNAs are sorted according to the ascending order of the maximum F_1_-score of BiokoP. For BiokoP, we report the maximum and minimum F_1_-scores of the set of solutions for each RNA. BiokoP finds a better solution than pKiss for 84 RNAs (among 198) and than McGenus for 103 RNAs while the optimal solutions found by pKiss and McGenus are better than the optimal solutions of the set generated by BiokoP for respectively 54 and 39 RNAs. The results show that BiokoP returns 61 better solutions compared to IPknot, while IPknot does not return better solutions compared to BiokoP. Returning several optimal solutions allows BiokoP to obtain the best solution more times than the other tools.

Finally, we observe that the gap between the minimum and the maximum F_1_-scores of BiokoP can be important. This shows that BiokoP returns a diversified set of optimal solutions.

#### Results by pseudoknot types

**Fig. 8:**
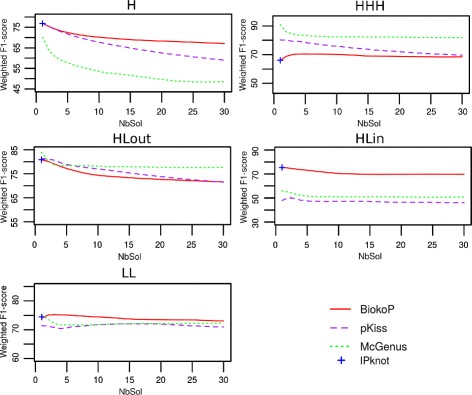
F_1_-score results in function of pseudoknot types. Weighted means of F_1_-scores of the structures predicted with BiokoP, pKiss, McGenus and IPknot on the different types of pseudoknotted RNAs (154 H, 3 HHH, 26 HLout, 4 HLin and 11 LL), in function of the number of solutions (NbSol)

Figure 8 reports the F_1_-score results in function of pseudoknot types and of the number of solutions returned. The results for the H-type pseudoknots are very similar to the results of the entire dataset, which is not surprising since the H-type is largely represented in it (154 among 198 RNAs). The HHH and HLout pseudoknot types are better predicted by McGenus, with weighted means of F_1_-scores around 84 and 79% respectively. However, for the HLin and LL types, BiokoP outperforms pKiss and McGenus with weighted means of F_1_-scores around 70% (HLin) and 75% (LL), whereas the weighted means of F_1_-scores of pKiss and McGenus are around 60 and 50% respectively for the HLin type and around 70%, for both tools, for the LL type. The results show that compared to IPknot, BiokoP obtains better F_1_-scores for the HHH and the LL pseudoknot types, and similar F_1_-scores for the other types when considering one solution returned.

The BOIP of BiokoP has been modeled to be able to predict any kind of pseudoknots. This is confirmed by the results obtained that are very homogeneous. Indeed, the F_1_-scores of BiokoP are never lower than 70% for any number of solutions returned. This is not the case for pKiss and McGenus, for which we can observe that the results depend greatly on the pseudoknot type. In particular, they obtain F_1_-scores around 50% for the HLin type. Since the datasets of some pseudoknot types are small (3 HHH, 26 HLout, 4 HLin, 11 LL and 154 H), further experiments need to be done to confirm the results.

Finally, when one wants to predict a secondary structure of an RNA, there is generally no information about the pseudoknot type. Therefore, it is better to use BiokoP, which allows to predict structures as close as possible to the real ones, regardless of the pseudoknot type.

#### Results on pseudoknot-free RNAs

In order to determine if BiokoP is able to predict pseudoknot-free structures of RNAs, we evaluated it on our dataset of pseudoknot-free RNAs and compared the obtained results with those obtained by pKiss, McGenus, IPknot and also RNAsubopt which is a tool dedicated to pseudoknot-free RNA prediction.

**Fig. 9:**
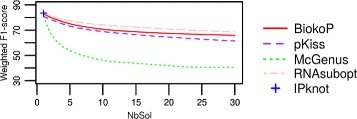
F_1_-score results on pseudoknot-free RNAs. Weighted means of F_1_-scores of the structures predicted with BiokoP, pKiss, McGenus, RNAsubopt and IPknot on pseudoknot-free RNAs, in function of the number of solutions (NbSol)

In Fig. 9, we present the weighted means of F_1_-scores obtained by BiokoP, pKiss, McGenus, RNAsubopt and IPknot on the pseudoknot-free RNAs, in function of the number of returned solutions. BiokoP, pKiss and RNAsubopt give comparable results, showing that BiokoP and pKiss are both able to predict pseudoknot-free structures, unlike McGenus. We expected a bigger difference with RNAsubopt since this tool is made to generate only pseudoknot-free structures, but finally, it seems that BiokoP and pKiss do not suffer from a bias due to their purpose of returning pseudoknotted structures.

#### Illustration on some examples of RNAs

**Fig. 10:**
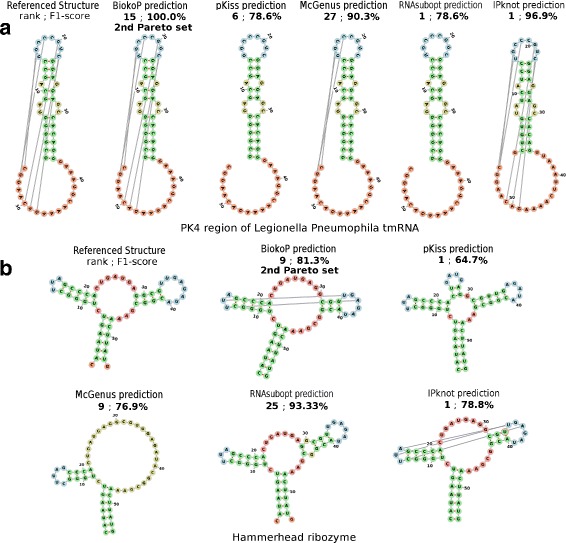
Evaluation of BiokoP on some examples of RNAs. Reference structures and best predictions of BiokoP, pKiss, McGenus, RNAsubopt and IPknot for 30 solutions returned, of (**a**) the pseudoknot region PK4 (H type) [35] of the Legionella Pneumophila tmRNA (PKB70) and (**b**) a pseudoknot-free hammerhead ribozyme (RFA_00393) [37]. For each structure is displayed the rank and the F_1_-score. The views were obtained with the RNA visualization tool forna from the ViennaRNA Web Service [36]

In order to give an overview of the predictions of BiokoP, we tested it on two known RNAs, for 30 solutions returned. We evaluated BiokoP with the pseudoknot region PK4 (H type) [35] of the Legionella Pneumophila tmRNA. The referenced structure of this RNA and the best solution according to the F_1_-score returned by BiokoP, pKiss, McGenus, RNAsubopt and IPknot are presented in Fig. 10a. The views are obtained with forna [36], the RNA visualization tool from the ViennaRNA Web Service. We observe that the exact referenced structure is found by BiokoP at rank 15, in the second Pareto set. McGenus and pKiss find the global structure, but miss in part or completely the pseudoknot. RNAsubopt finds the same structure than pKiss. IPknot predicts the exact pseudoknot and the exact referenced structure except for one different base pair. All the tools have a high F_1_-score for this RNA, but BiokoP and IPknot are the only ones that succeed in predicting the exact pseudoknot. Furthermore, BiokoP is the only one that predicts the exact referenced structure.

We also study the results of BiokoP on a pseudoknot-free hammerhead ribozyme [37] (Fig. 10b). This particular structure, composed of three helices, is much studied for the understanding of the structure-function relation of RNAs. The best solution returned by BiokoP has a F_1_-score of 81.3% and is returned at rank 9, in the second Pareto set. BiokoP finds the two first helices and some base pairs of the third one, but predicts an extra pseudoknot of two base pairs. pKiss and McGenus find the two first helices of the RNA. However, pKiss predicts an hairpin that does not exist in the referenced structure and McGenus does not find any base pair of the third helix. RNAsubopt finds the two first helices and some base pairs of the third one. The structure predicted by IPknot is identical to the one predicted by BiokoP, but its pseudoknot possesses an additional base pair. This example supports the previous results on the pseudoknot-free RNA dataset. As RNAsubopt predicts structures without pseudoknots, its results are better than the ones obtained by the other tools which can predict extra pseudoknots. However, the best solution of RNAsubopt is predicted at rank 25 which is far compared to the one of BiokoP.

### Execution time

The complexity of solving optimally integer programs is exponential with respect to the number of variables, which depends mainly on the length of the sequence. Moreover, we deal here with multi-objective programming which increases the number of BOIPs to solve and their number of variables. As estimation of the time of BiokoP, the prediction of 30 secondary structures for an RNA of 30 nucleotides on Debian OS with 24 Intel Xeon CPU X5660 (2.8 GHz) and 64Go RAM took, in average, 22 s. For the pseudoknot region PK4 [35] of the Legionella Pneumophila tmRNA (PKB70) of 55 nucleotides, BiokoP took 5min30.

## Discussion

We developed an original generic algorithm to solve BOIPs and to return several optimal solutions (the exact Pareto set) and several sub-optimal solutions (the sub-optimal Pareto sets). We proposed a BOIP that combines two models of RNA secondary structure prediction with pseudoknots, namely the MEA and the MFE models. The implementation of our algorithm with the proposed BOIP has led to a tool called BiokoP (Bi-objective programming pseudoknot Prediction).

Since our method is based on multi-objective optimization, BiokoP returns several sets of solutions (the *k*-best Pareto sets). Those Pareto sets are returned in an optimal order. However, all the solutions belonging to a Pareto set are not comparable and hence cannot be meaningfully ordered within the set, whereas the tools of the literature return solutions which are ordered optimally. Hence, comparing the results of BiokoP and of the tools of the literature raises some difficulties and, in multi-objective optimization, defining pertinent performance metrics for the Pareto sets is a subject of research [38]. We have chosen to rank the solutions within each Pareto sets assuming that a solution is better if it optimizes equally the two objectives, i.e., if the solution is close to the diagonal. This ranking allows us to show the benefits of combining Mod1 and Mod2. Indeed, the experiments show that for the first ranked solution returned, BiokoP is more likely to return the real structure than Mod1 and Mod2 (Fig. 5). The experiments also show that this combination allows to obtain homogeneous F_1_-scores compared to the other tools regardless of the number of solutions returned, the type of pseudoknots and the presence or not of pseudoknots (Figs. 6, 8, and 9).

However, this ranking shows some limitations. It is illustrated by the results detailed by pseudoknot types, where we observe that the solution giving the best F_1_-score is not always the first one (Fig. 8). To have a better idea of the global quality of the overall first Pareto set, we decided to compare it versus the optimal solution of the other tools, by studying the best and the worst structures (according to the F_1_-score) of the first Pareto set. The results show that in most cases, BiokoP finds more often better solutions than the other tools (Fig. 7).

The numerous experiments we performed show:

- that the combination of Mod1 and Mod2 is relevant: BiokoP returns all the real structures found by Mod1 and Mod2 as optimal solutions and is able to find more real structures (in optimal or in sub-optimal Pareto sets) than the two models alone;
- and that BiokoP, compared to the literature, achieves the best compromise between the number of times the best solution (with the highest F_1_-score) is found and the global quality of predictions.

## Conclusion

In this paper, we provide an original approach for predicting RNA secondary structures with pseudoknots that is based on the computation of several optimal and several sub-optimal solutions with respect to two different models. Our method is based on integer programming approach, that presents the advantage to be more flexible compared to the dynamic programming approach usually used in the RNA secondary structure prediction. In order to combine the two models, we developed a bi-objective integer program (BOIP), and proposed a generic novel algorithm to compute the sets of optimal and sub-optimal solutions for any BOIP. We applied it to the prediction of RNA secondary structures with pseudoknots, obtaining the software BiokoP. The experimental tests performed have confirmed the importance of considering sub-optimal solutions in addition to optimal ones. We have shown that our method successfully combines Mod1 and Mod2, taking benefit from the two models.

BiokoP was compared with other tools from the literature for RNA secondary structure prediction with pseudoknots that propose several solutions, namely pKiss [4] and McGenus [5]. BiokoP was also compared to IPknot [10]. IPknot predicts pseudoknotted structure, but no sub-optimal solutions. Finally, BiokoP was compared to RNAsubopt [22] which can predict only pseudoknot-free structures, but with sub-optimal solutions. Considering a set of 198 pseudoknotted secondary structures gathered from Pseudobase++ [20], BiokoP gives better F_1_-scores than pKiss and McGenus. We also show that BiokoP returns more time the best solution (with the higher F_1_-score) than pKiss, McGenus and IPknot, considering the optimal solutions. Even if BiokoP is made to predict pseudoknotted RNAs, the F_1_-score results for the dataset of pseudoknot-free RNAs are close to the results of RNAsubopt. The results of BiokoP are widely homogeneous regardless of the pseudoknot type or the presence or not of pseudoknots. Indeed, the F_1_-scores are always higher than 70% and regardless of the number of solutions returned, unlike pKiss and McGenus.

A drawback of our approach is the time complexity due to the need to optimally solve several integer programs. As stated before, this complexity is exponential, with respect to the length of the sequence. A perspective to decrease the execution time is to use parallelism. Indeed, due to its recursive shape, the generic algorithm we propose to solve BOIP is suitable to parallelization. Another idea to decrease the complexity of the BOIP would be to add constraints when some information on the structure are known: base pairs, presence or not of pseudoknots, type of pseudoknots.

In this work, we combined two models inspired from the literature. A perspective is to combine better mono-criterion models in order to raise the global quality of the solutions found. We would first try to model the comparative approach with integer programming to propose a combination with one of the thermodynamic model. This would take benefit from the additional information brought by homologous sequences. Finally, another future work will consist in developing multi-objective algorithms in order to combine more than two models. Moving from two objectives to three will require to rethink the current algorithm in three dimensions. This would represent the first step to develop a multi-objective generic algorithm able to return sub-optimal Pareto sets for any IP with *n* objective functions.
